# Chemodynamics of Methyl Parathion and Ethyl Parathion: Adsorption Models for Sustainable Agriculture

**DOI:** 10.1155/2014/831989

**Published:** 2014-02-06

**Authors:** Noshabah Tabassum, Uzaira Rafique, Khaled S. Balkhair, Muhammad Aqeel Ashraf

**Affiliations:** ^1^Department of Environmental Sciences, Fatima Jinnah Women University, The Mall, Rawalpindi 46000, Pakistan; ^2^Department of Hydrology and Water Resources Management, King Abdulaziz University, Jeddah 22254, Saudi Arabia; ^3^Department of Geology, Centre for Research in Biotechnology for Agriculture, University of Malaya, 50603 Kuala Lumpur, Malaysia

## Abstract

The toxicity of organophosphate insecticides for nontarget organism has been the subject of extensive research for sustainable agriculture. Pakistan has banned the use of methyl/ethyl parathions, but they are still illegally used. The present study is an attempt to estimate the residual concentration and to suggest remedial solution of adsorption by different types of soils collected and characterized for physicochemical parameters. Sorption of pesticides in soil or other porous media is an important process regulating pesticide transport and degradation. The percentage removal of methyl parathion and ethyl parathion was determined through UV-Visible spectrophotometer at 276 nm and 277 nm, respectively. The results indicate that agricultural soil as compared to barren soil is more efficient adsorbent for both insecticides, at optimum batch condition of pH 7. The equilibrium between adsorbate and adsorbent was attained in 12 hours. Methyl parathion is removed more efficiently (by seven orders of magnitude) than ethyl parathion. It may be attributed to more available binding sites and less steric hindrance of methyl parathion. Adsorption kinetics indicates that a good correlation exists between distribution coefficient (Kd) and soil organic carbon. A general increase in Kd is noted with increase in induced concentration due to the formation of bound or aged residue.

## 1. Introduction

Sustainable agriculture demands high and good quality food production. Increase in agricultural base has become a challenge for the growers and farmers. This compels extensive use of insecticides that lead to growing accumulation of pollutants in environment over the last decade. The environment and human health are adversely affected by irrational and high pesticides use [1]. The toxicological and ecotoxicological effect is manifested as pesticides remain chemically active and rapidly broke down into other chemicals [2]. Pesticides when applied on crops get transported to various environmental compartments [3] like soil, plant, and water, while only a small part of the chemical stays in the area where it is applied.

Organophosphates have been detected in air, snow, fog, rainwater [4, 5], and in the pine needles in the mountains [6], miles away from the agricultural spraying area. Toxicity of organophosphates for nontarget organisms has also been the subject of extensive research [7]. Organophosphates are extensively used in China, Colombia, and Pakistan. Use of chemicals to control pests is increasing at the rate of 25% a year [8] in Pakistan.

Organophosphates are esters of phosphoric acid and exist in two forms, Thion and Oxon [9]. Parathions (methyl and ethyl) are a group of highly toxic compounds used extensively in agricultural crops especially cotton, soybean, corn, wheat, alfalfa, vegetables, fruit trees, and domestic activities [10] leading to different hazards.

Methyl parathion, C_8_H_10_NO_5_PS [11], also known as metaphos, is a broad-spectrum agricultural insecticide and acaricide that is released to the environment primarily through spraying using aircraft or ground spray equipment [12]. Methyl parathion is rapidly removed from the atmosphere [13] by wet and dry deposition and forms bound residues restricting its movement in soils [14–16], where its adsorption is influenced by organic matter and CEC of the soil [17]. However, its mobility and leaching into the soil-water system is affected by pH. Methyl parathion when introduced into the environment is degraded by hydrolysis, photolysis, or microorganisms, whereas degradation appears to be significantly retarded [18, 19] when its concentration is high, as in bulk disposal and spills.

Ethyl parathion, C_10_H_14_NO_5_PS, also known as thiophos, has little or no potential for groundwater contamination [11]. The major metabolites of ethyl parathion are amino parathion and 4-nitrophenol. However, in soils that have received multiple applications, 4-nitrophenol is the only metabolite and rate of degradation is faster. Soil act as a buffer and offer degradation potential for the stored pollutants with the help of soil organic carbon [20]. Pesticides also bind to soil particles thus reducing chemical availability and transportation to different environmental compartments. Chemodynamics of pesticides is generally considered to be effectively controlled through adsorption process by offering high adsorption capacity, extra ordinary surface area, and microporous structure of adsorbents.

To remediate the adverse effects and chemical accumulation of active metabolites of applied insecticides in soil fields, different control methods are in use. Solid-phase adsorption is one of the most efficient technologies for the treatment of pesticide [21].

The adsorption of organophosphorus pesticides onto activated carbon has attracted many researchers due to its high removal efficiency [22, 23], but high cost inhibits its application on a large scale [24].

To overcome these and other limitations associated with commercial adsorbent materials, the researchers continue their work to find out more economical and easily available materials [25] to be used as potential adsorbents.

Knowing the fact that sorption of organic chemicals to soil is a process that can affect mobility, degradation, and toxicity by reducing availability, the present investigation is designed with the following objectives:

- explore the use of most abundantly available soil as natural adsorbent for the removal of organophosphate pesticides;
- quantify the fate and transport process of organophosphates for understanding their behavior in the environment;
- determine the factors affecting binding of pesticide with soil through batch adsorption experiment.

Pakistan is a developing country with agro-based economy. Its life line and development rest on sustainable agricultural practices. Improving soil conditions of agricultural fields can ensure best growing conditions and can also offset the adverse effects of applied pesticides; for example, organic matter in soil has multiple functions. It revolves nutrient storage, improves soil structure, maintains tilt, minimizes erosion, and binds the unwanted chemical to be removed thereafter.

The present study will facilitate the prediction of the exposure level of humans and nontarget organisms to organophosphate pesticides and its active ingredients. The development of low cost soil adsorbent will suggest a pest control product for environmental remediation and sustainable agriculture.

## 2. Materials and Methods

Two different sampling areas of Jhelum, that is, agricultural (wheat) and barren (suburbs of wheat), were selected for sampling with the objective to represent varying organic matter. Topsoil (4-5 inches) 90 samples from agricultural and 60 of barren area were collected in X and zigzag pattern, respectively. Composite sieved (2 mm) soil sample of each type was prepared by mixing the subsoil samples in agate mortar and pestle. pH and temperature were noted on site.

Each soil sample was analyzed for physicochemical parameters of pH, bulk density, electrical conductivity, and organic matter content following standard methods. The pH and EC of all solutions were recorded by pH (inoLab pH 720) and conductivity meter (con-500, Cyberscan), respectively. Color of samples was observed using Munsell color chart. Results of physicochemical parameters are summarized in Table 1.

The physicochemical characteristics of oil samples reveal that agricultural fields have relatively lower pH than barren. It may be related to the higher organic content due to more fulvic and humic acid. The range is generally alkaline for both classes of soil. Pakistani soil is mostly alkaline in nature ranging from 7.5 to 8.5 [26].

The low value of bulk density is indicative of higher organic matter content and large pore size [27]. Soil with higher content of organic matter is more porous and has relatively low bulk density [28].

EC of the soil sample decreases with decrease in % age organic matter (see Table 1). It may be attributed to the fact that ionic concentration is greater in alkaline soils [29] and the higher the ionic species, the higher the conductivity [30].

### 2.1. Batch Adsorption

Nine series of batch experiments for each pesticide were conducted as a function of time to determine the percentage concentration of the pesticide removed by adsorption on each soil. The following general procedure for a batch experiment was adopted.

Aqueous solution of known concentration of the pesticide was induced to fixed mass (5 g) of soil adsorbent, adjusted at known pH at room temperature. Solution pH was adjusted to the desired value (pH 4, 7 and 10) by adding sodium acetate and acetic acid (pH 4), 0.1 M NH_3_, and NH_4_OH for pH 7 to 10 solutions [31].

The mixture was allowed to shake on Isothermal shaker (Lab-Companion SK-300). After regular contact time interval (one hour), pesticide was extracted using equimolar solvent mixture of acetone and n-hexane. The extracted aliquot was run on UV-Visible spectrophotometer (UV1601, Shimadzu) to determine the absorbance of the solution against blank. The concentration was calculated from standard calibration curve. The process continued till equilibrium was attained between adsorbate and adsorbent.

The same procedure was repeated for varying adjusted pH (4, 7 and 10) and variable induced concentration in *μ*g/L (10, 30 and 50) for each selected pesticide.

The percentage removal of methyl parathion and ethyl parathion by different soils at equilibrium is calculated using the following mass balance equation:

(1) $$\begin{array}{c} q_{e}=\frac{C_{i}-C_{e}}{S}, \end{array}$$

where *q*_*e*_ is amount (in *μ*g/kg) of the pesticide removed, *C*_*i*_ is initial concentration of pesticide in solution (*μ*g/L), and *C*_*e*_ is equilibrium concentration of pesticide in solution (*μ*g/L).

The dose concentration *S* is expressed as *S* = *m*/*v*, where *v* is initial volume of pesticide solution used and *m* is mass of soil used.

*K*_*d*_ and *K*_oc_ were also calculated using the following equations:

(2) $$\begin{array}{c} K_{d}=\frac{\text{amount}\;\text{of}\;\text{pesticide}\;\text{in}\;\text{adsorbent}}{\text{amount}\;\text{of}\;\text{pesticide}\;\text{in}\;\text{solution}}, \end{array}$$

(see [32]),

(3) $$\begin{array}{c} K_{\text{oc}}=\frac{K_{d}}{\text{OC}}, \end{array}$$

(see [33]).

*K*_*d*_ is the distribution coefficient so *K*_*d*_ = *X*/*S*, where *X* is the amount of adsorbent and *S* is the amount of pesticide in solution. *K*_oc_ is the distribution coefficient of organic carbon and OC is the organic carbon.

### 2.2. Kinetic Studies

The adsorption kinetics was computed to optimize the appropriate correlation for equilibrium adsorption behavior. Rate was determined through application of first order, pseudo-first-order [34], pseudo-second-order [35], and intraparticle diffusion [36].

### 2.3. Adsorption Models

Adsorption models of Freundlich and Langmuir [37] are commonly used to describe the adsorption process. Equations are tabulated in Table 2.

## 3. Results and Discussion

### 3.1. Effect of pH

**Figure 1:**
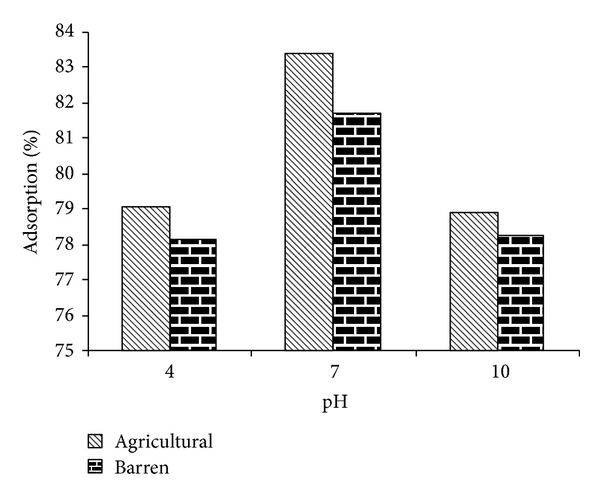
Effect of pH on %-adsorption of methyl parathion.

**Figure 2:**
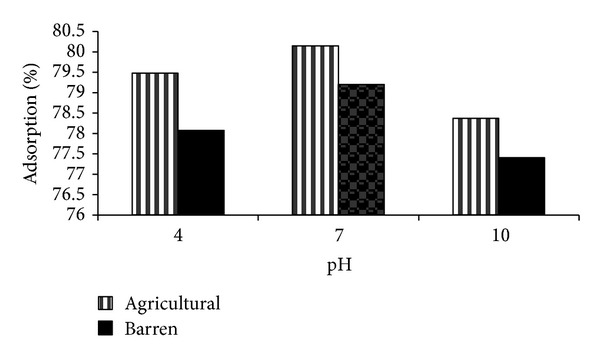
Effect of pH on %-adsorption of ethyl parathion.

The effect of different pH (4, 7 and 10) on the adsorption of methyl parathion and ethyl parathion by different soil samples is studied. The results are graphically presented in Figures 1 and 2.

It is observed that pH has a momentous effect in adsorption capacity. In moving from pH 4 to 7, an increase in methyl parathion adsorption followed by a decrease at pH 10 is observed for agricultural and barren soil. The presence of hydronium ions on the adsorbent surface at lower pH may enhance the interaction of pesticide molecules with the binding sites of adsorbent material. It is further suggested that carbonaceous functional groups are dissociated at different pH values and consequently take part in the sorption process.

Same trend is noted for adsorption of ethyl parathion on both soil types in terms of variable pH (see Figure 2).

The present study behavior of organophosphates (parathion) is in contrast to Lindane and Carbofuran [38] reporting that adsorption increases with increase in pH of neutral molecules.

Decrease in percent adsorption with time is accompanied by a reduction in the adsorption capacity while extending to basic pH in both soil types. The reduction in adsorption capacity at higher pH is also reported by other authors [39]. It may also be attributed to the lesser adhesive forces between adsorbate and adsorbent than the cohesive forces of the adsorbate (due to alkaline soil and adjusted alkaline media).

The study concludes that pH 7 is optimum evident to both pesticides, showing maximum removal efficiency for methyl and ethyl parathions as 83% and 80% for agricultural soil, whereas 82% and 79% for barren soil.

It reflects preference of organic matter content in agricultural soil for adsorption; the higher is the organic content, the more is the adsorption. Soil high in organic matter and clay are more adsorptive than coarse sandy soil because a clay or organic soil has more particle surface area or more sites into which pesticide can bind [40]. The closeness in percent adsorption on two types of soil at pH 7 (neutral) is highly encouraging as sustainable agricultural approach suggests that little or no modification is required in soil characteristics for optimum removal of parathion.

### 3.2. Effect of Concentration

**Figure 3:**
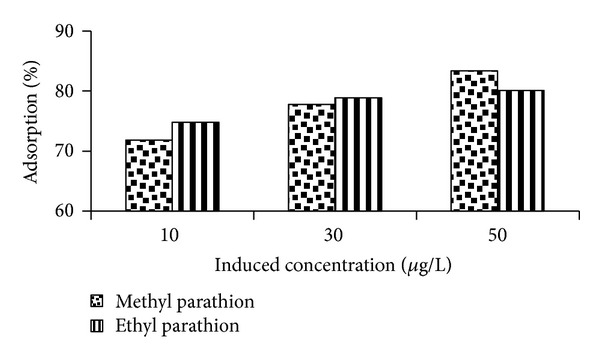
Effect of induced concentration on %-adsorption of methyl parathion.

**Figure 4:**
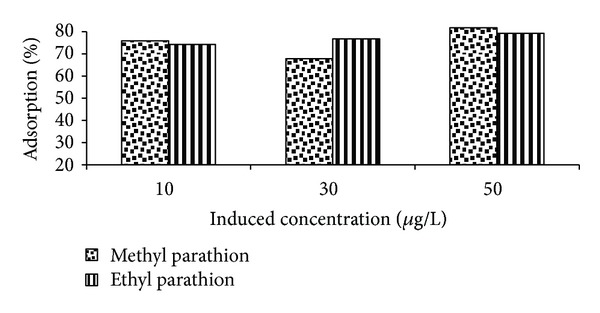
Effect of induced concentration on %-adsorption of ethyl parathion.

In order to study the effect of concentration on adsorption, batch experiment was administered at induced pesticide concentration of 10, 30, and 50 *μ*g/L for each soil type. The results are presented in Figures 3 and 4.

It is noted that adsorption potential of agricultural soil for removal of methyl and ethyl parathions is found to be 72%, 78%, and 83% and 75%, 79%, and 80%, respectively, on increasing the concentration from 10 through 30 to 50 *μ*g/L (see Figure 3).

As reported in the literature the maximum loading capacity of the adsorbent and the rate of adsorption were found to increase with increase in the pesticide initial concentration [24].

A different behavior is depicted by barren soil field samples for the removal of methyl parathion with change in concentration. An initial decrease of 8 orders followed by 14 orders increase in adsorption is observed in moving from 10 *μ*g/L to 50 *μ*g/L (see Figure 4). This peculiar feature of methyl parathion adsorption is affected by the chemical properties of the pesticide [41].

The general lower adsorption behavior on barren soil is demonstrated by soil parameters like higher moisture content, significantly lower EC, and high density allowing less number of sites available.

On the other hand, barren soil behaves similarly to agricultural soil for the removal of ethyl parathion showing a gradual increase in adsorption with a regular increase in concentration. This is due to the fact that increased concentration provides necessary driving force to overcome the resistances to the mass transfer of pesticide between aqueous and solid phase. This behavior is comparable and supported by other studies [38].

The study concludes that optimum adsorption takes place at higher induced concentration (50 *μ*g/L). However, the induced concentration on average impact slightly shows more adsorption for methyl parathion than ethyl parathion. It may be due to insignificant structural difference between two pesticides.

### 3.3. Effect of Contact Time

Batch experiment was conducted with regular intervals of time in order to determine the equilibrium between adsorbate and adsorbent.

**Figure 5:**
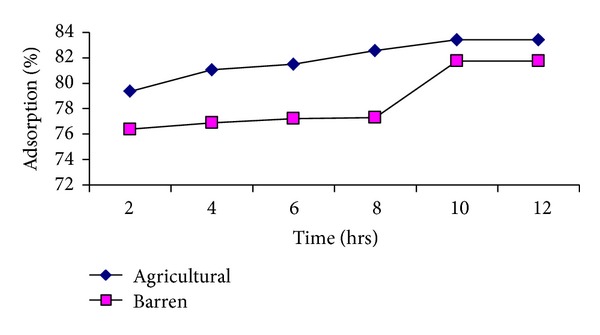
Effect of contact time on %-adsorption of methyl parathion.

**Figure 6:**
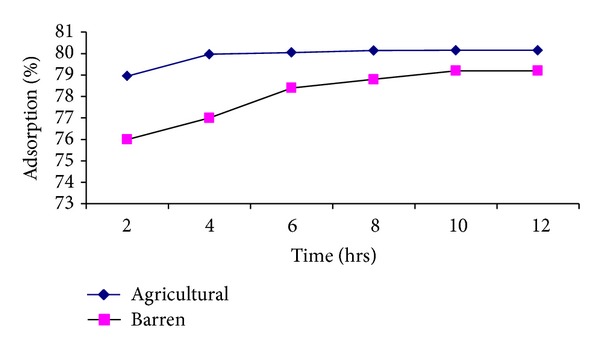
Effect of contact time on %-adsorption of ethyl parathion.

It is generally observed that adsorption increases with increase in contact time for both pesticides. The removal was rapid in early stages and finally attained almost constant value for longer contact time (see Figures 5 and 6). Obviously, the initial high adsorption rate is due to the abundance of free binding sites [24].

It is interesting to note that the first adsorption equilibrium for both pesticides is attained in 10 hours time. This is also supported by other studies. However, the rate of adsorption follows a very slow increase for barren soil till equilibrium. Agricultural soil shows a rapid increase in adsorption for the first 6 hours followed by almost the same adsorption rate till equilibrium.

### 3.4. Adsorption Kinetics

The average values of the adsorption kinetic parameters for both pesticides on two soil types are tabulated in Table 3.

It can be seen that pseudo-second-order kinetic fits the adsorption data equally best for both soil types and both pesticides with correlation coefficient *R* ^2^ (0.999) at optimum operating conditions of pH and induced concentration.

Intraparticle diffusion kinetics also provides a good description of sorption data. A set of correlation coefficients of 0.979, 0.918 and 0.976, 0.961 is comparable for methyl and ethyl parathions on agricultural and barren soils, respectively.

It can be seen from Table 3 that distribution of methyl parathion in agricultural soil samples is comparatively higher than ethyl parathion. This is also in conformity with higher adsorption of the former on agricultural soil.

The sequence of distribution constant as a function of pH follows pH 7 < pH 4 < pH 10 on agricultural and barren soil for both pesticides. On further investigation, a direct relation of *K*_*d*_ and *K*_oc_ is found verifying the trend that agricultural soils are relatively proven to be better adsorbent and work optimally at pH 7. Direct relation of *K*_*d*_ and *K*_oc_ depicting higher values for agricultural than barren soil is reported by other researchers [42] (see Table 4).

### 3.5. Adsorption Isotherms

Both Freundlich and Langmuir isotherms are best fit for experimental data. The magnitude of *K*_*f*_ (see Table 5) shows relatively good adsorption capacity. Dada et al. reported *K*_*f*_ value of 7.61 mg/g for adsorption of Zn onto modified rice husk [43]. Good fit of Freundlich isotherm describes that the adsorbent surface is heterogeneous in nature [44].

On the other hand, Langmuir also holds best sorption data with average *R* ^2^ (0.998). Langmuir model suggests formation of a monolayer adsorbate on the outer surface of the adsorbent and equilibrium distribution between the solid and liquid phases [45].

## 4. Conclusions

The present study concludes with the following:

- the batch adsorption experiment provides an efficient, convenient, and simple method for the removal of selected pesticides attaining equilibrium in 10 hours;
- the parameters of paramount significance are found to be organic matter content, pH and induced concentration, optimum being higher organic matter, pH 7, and higher (50 *μ*g/L) induced concentration for the removal of both pesticides;
- methyl parathion is found to be more effectively removed than ethyl parathion due to lower molecular weight and less steric hindrance;
- pseudo-second-order, intraparticle diffusion, Langmuir and Freundlich models explain the experimental data to the best fit.

The authors propose that adsorption attains equilibrium between adsorbate and adsorbent upon contact of 10 hours. The goodness of Langmuir suggests monolayer adsorption and nature of adsorbent (soil) is determined to be heterogeneous. It also reveals that pores are not uniformly distributed. kinetics reveals that pseudo-second-order is in good agreement for agricultural soil samples suggesting its dependence on concentration of organic content. On the other hand, intraparticle diffusion is found to be equally appropriate for both soils suggesting that diffusion is not characteristics of physical characteristics of soil and pore size is the same in both soil samples.

## Figures and Tables

**Table 1:** Physiochemical analysis of soil fields.

Sample fields	Wheat field (WF I)	Wheat field (WF II)	Wheat field (WF III)	Barren field (BF I)	Barren field (BF II)
pH	7.42	7.61	7.57	8.75	8.78
Electrical conductivity µS at 25°C	73.2	54.4	54.8	37.4	36.1
Bulk density g/cm^3^	1.33	1.36	1.37	1.5	1.6
Organic content (%)	4.2	4.4	4.5	3.1	3.2
Moisture content (%)	3.7	2.33	3.5	5.5	5.1

**Table 2:** Adsorption models along with their parameters.

Isotherms	Linear expression	Plot	Parameters
Langmuir (1918)	$\frac{C_{e}}{q_{e}}=\frac{1}{q_{m}K_{L}}+\frac{C_{e}}{q_{m}}$	$\frac{C_{e}}{q_{e}}$ v *C*_*e*_	$q_{m}=\frac{1}{\text{slope}}$
			$K_{L}=\frac{\text{slope}}{\text{intercept}}$

Freundlich (1906)	$\log q_{e}=\log K_{F}+\frac{1}{n}\log C_{e}$	log⁡*q*_*e*_ v log⁡*Ce*	$n=\frac{1}{\text{slope}}$
			*K*_*F*_ = Antilog (intercept)

Pseudo-first-order	$\log\left(q_{e}-q_{t}\right)=\log q_{e}-\left(\frac{k_{1}}{2.303}\right)t$	log⁡(*q*_*e*_ − *q*_*t*_) v *t*	*k*_1_ = slope
			*q*_*e*_ = Antilog (intercept)

Pseudo-second-order	$\frac{t}{q_{t}}=\frac{1}{k_{2}q_{e}^{2}}+\left(\frac{1}{q_{e}}\right)t$	$\frac{t}{q_{t}}$ v *t*	*q*_*e*_ = slope
			*h* = intercept
			$k_{2}=\frac{\text{intercept}}{{\left(\text{slope}\right)}^{2}}$

Intraparticle diffusion	*q*_*t*_ = *k*_ip_ *t* ^0.5^ + *C*	*q*_*t*_ v *t*	*k*_ip_ = slope
			*C* = intercept

**Table 3:** Kinetic models for methyl parathion and ethyl parathion.

Soil type	Kinetic models	Parameters	Methyl parathion	Ethyl parathion
		*K*_1_	−0.0011	−0.0011
Agricultural	Pseudo-first-order	*q*_*e*_	2.896	2.8903
		*R* ^2^	0.402	0.393

		*K*_1_	−0.0011	−0.0011
Barren	Pseudo-first-order	*q*_*e*_	2.8813	2.880
		*R* ^2^	0.3634	0.4023

		*K*_2_	0.1387	0.1295
Agricultural	Pseudo-second-order	*q*_*e*_	0.0014	0.0011
		*R* ^2^	0.999	0.999

		*K*_2_	0.0181	0.0255
Barren	Pseudo-second-order	*q*_*e*_	0.1059	0.0011
		*R* ^2^	0.5486	0.999

		*A*	0.0098	0.0105
Agricultural	Intraparticle diffusion	log⁡*K*_id_	1.8802	1.8925
		*R* ^2^	0.979	0.918

		*A*	0.0064	0.0167
Barren	Intraparticle diffusion	log⁡*K*_id_	1.8716	1.8763
		*R* ^2^	0.976	0.961

*q*_*e*_ is in µg/L.

**Table 4:** *K*_*d*_ and *K*_oc_ values of agricultural and barren soil samples.

	*K*_*d*_			*K*_oc_		
	pH 4	pH 7	pH 10	pH 4	pH 7	pH 10
Methyl parathion						
Agricultural	48.966	63.33	42.500	3726.71	3804.80	3079.71
Barren	44.516	51.429	42.500	3548.23	3632.48	3413.11

Ethyl parathion						
Agricultural	63.333	66.957	53.585	589.37	851.92	259.78
Barren	3.375	3.474	3.310	288.50	296.94	282.91

**Table 5:** Adsorption isotherm for methyl parathion and ethyl parathion.

Adsorption isotherm	Soil types	Parameters	Methyl parathion	Ethyl parathion
Freundlich	Agricultural	*n*	−1.606	−1.597
		*K*_*f*_	3.25	3.257
		*R* ^2^	0.999	0.999
	Barren	*n*	−1.602	−1.65
		*K*_*f*_	3.325	3.332
		*R* ^2^	0.999	0.999

Langmuir	Agricultural	*b*	−3.325	−3.271
		*Q*_*o*_	0.335	0.322
		*R* ^2^	0.999	0.998
	Barren	*b*	−3.161	−3.512
		*Q*_*o*_	0.282	0.332
		*R* ^2^	0.998	0.995
